# Breast cancer risk factor knowledge among nurses in teaching hospitals of Karachi, Pakistan: a cross-sectional study

**DOI:** 10.1186/1472-6955-5-6

**Published:** 2006-09-19

**Authors:** Faiza Ahmed, Sadia Mahmud, Juanita Hatcher, Shaista M Khan

**Affiliations:** 1 Division of Epidemiology and Biostatistics, Department of Community Health Sciences, The Aga Khan University, Karachi, Pakistan; 2 Research Development Office, Department of Community Health Sciences, The Aga Khan University, Karachi, Pakistan; 3 Department of General Surgery, The Aga Khan University, Karachi, Pakistan

## Abstract

**Background:**

Breast cancer is the most common cancer among women in both the developed and the developing world. The incidence of breast cancer in Karachi, Pakistan is 69.1 per 100,000 with breast cancer presentation in stages III and IV being common (≥ 50%). The most pragmatic solution to early detection lies in breast cancer education of women. Nurses constitute a special group having characteristics most suited for disseminating breast cancer information to the women. We assessed the level of knowledge of breast cancer risk factors among registered female nurses in teaching hospitals of Karachi. We also identified whether selected factors among nurses were associated with their knowledge of breast cancer risk factors, so that relevant measures to improve knowledge of nurses could be implemented.

**Methods:**

A cross-sectional survey was conducted in seven teaching hospitals of Karachi using stratified random sampling with proportional allocation. A total of 609 registered female nurses were interviewed using a structured questionnaire adapted from the Stager's Comprehensive Breast Cancer Knowledge Test. Knowledge of breast cancer risk factors was categorized into good, fair and poor categories. Ordinal regression was used to identify factors associated with risk knowledge among nurses.

**Results:**

Thirty five percent of nurses had good knowledge of risk factors. Graduates from private nursing schools (aOR = 4.23, 95% CI: 2.93, 6.10), nurses who had cared for breast cancer patients (aOR = 1.41, 95% CI: 1.00, 1.99), those having received a breast examination themselves (aOR = 1.56, 95% CI: 1.08, 2.26) or those who ever examined a patient's breast (aOR = 1.87, 95% CI: 1.34, 2.61) were more likely to have good knowledge.

**Conclusion:**

A relatively small proportion of the nursing population had good level of knowledge of the breast cancer risk factors. This knowledge is associated with nursing school status, professional breast cancer exposure and self history of clinical breast examination. Since only about one-third of the nurses had good knowledge about risk factors, there is a need to introduce breast cancer education in nursing schools particularly in the public sector. Continuing nursing education at the workplace can be of additional benefit.

## Background

Globally, breast cancer is the most common cancer among women [1]. Between 1975–1990, Asia and Africa have experienced a more rapid rise in the annual incidence rates of breast cancer than North America and Europe [2]. Karachi Cancer Registry, the only population based cancer registry in Pakistan, reports breast cancer as the most common cancer (34.6% of cancer cases) among females. The age-standardized incidence rate (to the world population) was 69.1 per 100,000 averaged over the years 1998–2002, the highest recorded rate of breast cancer in Asia [3]. Similarly in Lahore, another major city of Pakistan, breast cancer was the most common female cancer [4].

Pakistan faces a high burden of breast cancer disease with late stage presentation being a common feature. It has been seen that more than half of the patients present in advanced stages (stages III and IV) [5-12]. Regular clinical breast examination and mammography of women according to the internationally accepted guidelines can result in down-staging of breast cancer of asymptomatic women [3,13]. However, there are no national screening programs for breast cancer in Pakistan. In the Pakistani context, educating the women about the risks of breast cancer constitutes a first step towards early detection of breast cancer, so that women would be able to judge their risk and take relevant measures.

The important resources of dissemination of breast cancer knowledge to women are the health-care professionals, educational institutions and media. Among the healthcare professionals, female nurses comprise the group most suited for this purpose. In Pakistan a substantial number of nurses are women [14] and culturally, women patients are reluctant to go to male health care providers for problems such as breast diseases [5].

The nurses can play an important role in educating women through specially designed educational programs in the clinical setting, as well as, through community outreach strategies that suit our social and cultural setting. In addition, they constitute an important source of information within their social networks [15]. Since the nurses can have a major influence on the behavior of our women, they need to be knowledgeable themselves about breast cancer risk factors and the importance of early detection through screening.

Studies in the developing countries show diverse results ranging from poor to good knowledge about breast cancer. Among the Nigerian nurses, about half were well-informed of two out of five risk factors [16]. Sixty percent Iranian nurses correctly identified family history as a risk factor for breast cancer, while smaller proportions knew about other risk factors [17]. Most of the Jordanian nurses were able to correctly answer the general breast cancer questions which included risk factor questions [18].

A hospital-based study in Lahore, Pakistan, reported good levels of knowledge about breast cancer risk factors and screening methods among doctors and nurses [19]. However, the knowledge was not objectively evaluated and hence valid conclusions about the level of breast cancer knowledge among this group cannot be made. The aim of this study was to objectively assess the level of knowledge regarding risk factors of breast cancer and to evaluate factors associated with this knowledge among female registered nurses working in teaching hospitals of Karachi.

## Methods

A cross-sectional survey was conducted in seven teaching hospitals of Karachi between July and September 2003. Karachi is the largest city of Pakistan, with a population estimated to be about 13 million.

Teaching hospitals were defined as hospitals linked with a Medical College and/or a School of Nursing and were staffed by at least 50 female registered nurses. There are four government and four private teaching hospitals in Karachi that fulfill the above criteria. The administration of one private hospital refused permission for conducting the survey. Hence the survey was conducted in seven teaching hospitals. The catchment area of these seven hospitals extends beyond Karachi to include other regions of Pakistan.

The target population comprised of registered female nurses working in different departments of the seven teaching hospitals in Karachi. The minimum nursing education requirement for inclusion in the study was diploma in general nursing. Any nurse with a past or current history of breast cancer was excluded because her knowledge level could have been influenced due to her experience and interaction with health care providers.

The sampling technique employed was stratified random sampling. The seven hospitals were considered as strata. List of registered nurses was obtained from each hospital, male nurses were excluded from the list, and a computer generated simple random sample of female nurses was selected. The identified nurses belonged to different departments and after taking an informed consent the interviews were conducted in the work place. Trained female interviewers conducted the interviews using a structured questionnaire, assessing breast cancer risk factor knowledge, and recorded the answers. On average it took about 20 minutes to complete the interview. If a selected nurse was not present or was busy, a later attempt was made to contact her. A maximum of three attempts were made to locate a nurse. In order to avoid contamination among nurses within a hospital, the survey for each hospital was completed in the shortest possible time. Also the brochure containing information about breast cancer and breast self-examination was distributed to the nurses of a given hospital after the completion of the survey in that hospital.

The sample size was calculated to estimate the proportion of nurses having adequate knowledge with 95% confidence level and 3% error bound. As estimates for proportion of nurses having adequate knowledge were not available for the region, we assumed that in each stratum 50% of the nurses had adequate knowledge in order to get the maximum sample size. Inflating the sample size by 20% for non-response a total sample size of 638 nurses was obtained. We used proportional allocation to allocate the total sample size to each stratum. In the 7 hospitals, we could interview 609 nurses yielding a response rate of 95.45%. Twenty nine nurses could not be contacted even after three visits. The high response rate in our study could be due to the fact that 92% of the nurses were interested in learning more about breast cancer.

The knowledge assessment tool included five questions from the Stager's Comprehensive Breast Cancer Knowledge Test (general knowledge sub-scale) [20]. Five additional questions were formulated by the principal investigator using international and national literary sources taking into account the local context. The knowledge assessment tool is reported in Table 1. Content validity [21] was established by expert opinion of a surgeon with extensive breast cancer experience (SK in the list of authors) and an epidemiologist with experience in cancer epidemiology (JH in the list of authors). The reliability of the instrument was assessed by calculating the Kuder-Richardson 20 (KR-20) as each individual item had a dichotomous response (Yes/No).

**Table 1:** Individual items and respective scores assessing knowledge of breast cancer risk factors with percentage of correct responses:

**Items**	**Correct answer**	**Score**	**Correct response %**
1. Breast cancer is a communicable disease	No	1	99.2
2. The irritation of a tight bra can over time cause breast cancer	No	1	59.4
3. In some women being overweight increases the risk of developing breast cancer	Yes	1	27.6
4. A woman who bears her first child after the age of 30 years is more likely to develop breast cancer*	**Yes**	**3**	**50.2**
5. Use of oral contraceptives increase a woman's risk of breast cancer	Yes	1	49.6
6. A hard blow to the breast may cause breast cancer later in life	No	1	24.6
7. Most breast lumps are cancerous	No	1	73.7
8. A woman, who has a first blood relative with breast cancer, is at higher risk of developing breast cancer*	**Yes**	**3**	**57.8**
9. Breast feeding increases the chance of breast cancer	No	1	96.4
10. Breast cancer can be a result of a curse/evil eye*	**No**	**2**	**94.9**
**Total**		**15**	

* Key items

In the assessment tool, three items were identified as key items on the basis of their relative importance. The three key items comprised of knowledge regarding family history of breast cancer [22], late age at first pregnancy [23] and myths about curse/evil eye being a contributory factor towards breast cancer. The first two are established risk factors for breast cancer and each were given a score of 3, the last is relevant as myths regarding disease development are common in the Pakistani society [24] and was given a score of 2. The remaining seven items were given a score of 1. The total score ranged from 0 to 15, which was categorized into good, fair and poor categories on the basis of the three keys items as follows:

• Nurses who did not answer any key item correctly can get a maximum score of 7 and were labeled as having **"poor knowledge"**.

• A nurse who answered only one key question correctly cannot score greater than 10. Accordingly scores from **8 to 10 **were classified as **"fair knowledge"**.

• The category **"good knowledge**" comprised of scores **11 to 15 **and corresponds to nurses who answered at least two key items correctly.

In addition to assessing the knowledge of breast cancer risk factors, demographic characteristics (age, marital status, income, education) and information regarding work history was recorded. The nurses were also asked about personal health history related to breast, history of breast cancer among family and friends, self-perceived breast cancer knowledge and potential sources of this knowledge.

Study approval was obtained from the Ethical Review Committee of the Aga Khan University. Permission was sought from each hospital administration to conduct the survey in the respective hospital.

Analysis was performed using SAS, version 8 [25]. Descriptive analysis was run for the independent variables and the outcome, that is, knowledge regarding breast cancer risk factors considered as an ordinal variable. The proportions of nurses having good, fair and poor knowledge, respectively, were estimated.

Ordinal regression using the cumulative logit model [26] was conducted to identify factors associated with the knowledge level. We performed ordinal regression on SAS using 'proc logistic'. Adequacy of the proportional odds assumption was assessed by the score test.

## Results

The mean age (standard deviation) of female registered nurses in our sample was 32 ± 8 years. Eighty three percent of the nurses had received basic level nursing education only including general nursing diploma alone or general nursing diploma with lady health visitor or with midwifery certification. Forty four percent of the nurses in our sample had attended a private school of nursing and 58% were employees of private hospitals at the time of the survey (Table 2).

**Table 2:** Personal characteristics of female nurses working in teaching hospitals of Karachi, Pakistan 2003 (n = 609)

**Variables**	**Numbers (%)**	**Mean (SD)**
Age (years)		32 (8)
Highest nursing education received:		
***General nursing diploma***	229 (38)	
***General nursing diploma & midwifery or General nursing diploma & lady health visitor***	274 (45)	
***Post-graduation and/or any diploma in any specialty***	75 (12)	
***Bachelor of science in nursing***	31 (5)	
Graduated from a private school of nursing	265 (44)	
Currently working in a private hospital	354 (58)	
Duration of work as a nurse (years)		9 (7)
Ever cared for a breast cancer patient	406 (67)	
Ever performed clinical breast examination on a patient	365 (60)	
Ever undergone a clinical breast examination	386 (63)	
Family history of breast cancer	49 (8)	
Friends/acquaintances history of breast cancer	60 (10)	
Self-perceived knowledge of breast cancer	590 (97)	
Interested in breast cancer education	563 (92)	

Thirty five percent of the nurses in our sample had good knowledge, 40% had fair knowledge while 25% nurses had poor knowledge of breast cancer risk factors. The reliability coefficient (KR-20) for the tool was 0.1 which is considered quite low.

Ninety-nine percent of the nurses in our sample correctly identified breast cancer as a non-communicable disease, 96% knew that breast feeding is not causative of breast cancer and 95% answered that evil eye has nothing to do with breast cancer. However, only about 28% of the nurses knew that in some women being overweight increases the risk of developing breast cancer (Table 1).

The proportional odds assumption for the ordinal regression analysis (cumulative-logit model) was satisfied (p-value = 0.69). Adjusting for other variables present in the final model (Table 3), nurses graduating from a private school of nursing were more likely to have good risk factor knowledge (aOR = 4.23; 95% CI: 2.93, 6.10) compared to nurses graduating from a public school of nursing. The odds of good risk factor knowledge were higher if the nurse had received a clinical breast examination (CBE) in the past (aOR = 1.56; 95% CI: 1.08, 2.26). Similarly a nurse was more likely to have good knowledge of risk factors if she had performed a breast examination (CBE) on a patient (aOR = 1.87; 95% CI: 1.34, 2.61). Ever having cared for a breast cancer patient was also associated with good risk factor knowledge (aOR = 1.41; 95% CI: 1.00, 1.99).

**Table 3:** Multivariable ordinal regression model for factors associated with good knowledge of breast cancer risk factors:

**Variables**	**Adjusted Odds Ratio**	**95 % Confidence Interval of Odds Ratio**
**School of nursing:**		
*Public(reference)*	1	
*Private*	4.23	2.93, 6.10
**Undergone a breast examination**:		
*Not undergone(reference)*	1	
*Undergone*	1.56	1.08, 2.26
**Performed breast examination on a patient**:		
*Not done(reference)*	1	
*Done*	1.87	1.34, 2.61
**Cared for a breast cancer patient**:		
*No(reference)*	1	
*Yes*	1.41	1.00, 1.99

Chi-square test statistic for score test = 2.36; p-value = 0.67

Chi-square test statistic for Likelihood ratio test = 146.64; p-value < 0.0001

## Discussion

Our study estimated that 35% of registered nurses in the teaching hospitals of Karachi had good knowledge of breast cancer risk factors. Nurses who graduated from a private nursing school or who have had professional breast cancer experience were more likely to have good knowledge.

The knowledge of breast cancer risk factors among the nurses of Karachi is low and is similar to that seen in other developing countries [16,17]. Our study sample comprised of a random mix of nurses working in various units of both public and private hospitals, who did not differ in their knowledge level. It is conceivable that risk factor knowledge is mostly acquired during classroom teaching compared to exposure at the workplace. The health care professionals work with patients so they are mainly exposed to symptoms and signs of disease and to treatment outcomes rather than to the development process of the disease especially for non-communicable diseases such as cancer. The low level of risk factor knowledge among nurses in the developing countries is suggestive of insufficient emphasis on the importance of primary prevention in the nursing curricula. In spite of rigorous efforts towards improving medical education in the developed countries, it has been realized that healthcare professionals including nurses are not adequately educated about cancer risk factors, risk assessment and cancer prevention [27].

Breast cancer risk factor knowledge among nurses is important so that they can provide appropriate screening recommendations to women with a high risk profile, especially in the Pakistani context where breast cancer screening is not a national phenomenon.

Nurses graduating from the private school of nursing were about 4 times more likely to have good knowledge of risk factors of breast cancer compared to nurses graduating from public school of nursing, implying relatively better educational standards of private schools of nursing. This finding is supported by the fact that some private institutions in Pakistan have instituted post-basic nursing education beyond the diploma level while the government sector does not have such programs [28]. However, at the diploma level the length of the educational process is similar in both the private and public institutions indicating a difference in the quality of education. This is an area which requires further research.

A nurse who had cared for a breast cancer patient or had performed clinical breast examination (CBE) on a patient during her nursing career was better informed of breast cancer risk factors. The association with workplace exposure seems to be consistent with the general opinion of nurses in Manchester, England who identified 'nursing patients' as the most important source of cancer information [29].

Our study indicates that a nurse who had ever received a breast examination by a health-care professional was more knowledgeable about breast cancer risk factors. Among nurses employed in the Public Health Service in Singapore, breast cancer risk factor and screening knowledge was not associated with receiving a clinical breast examination in the past year [30]. In our study, the breast examination was done as part of the general examination at the start of employment or of antenatal checkup for most of the nurses and it could be that the nurse was informed by the health care provider about the risk factors of breast cancer during the examination process. There is a need to assess the breast cancer counseling practices of health care providers.

One of the private hospitals which were initially selected for the study turned down our request for the survey. It is unlikely that our results may have been affected by the exclusion of these nurses because we had similar representation from the private institutions included in our study in terms of the health services offered, the recruitment of nurses, academic activities and the catchment population.

The questionnaire for our study was adapted from a validated questionnaire after modification. Content validity was established through peer review. In addition, construct validity of our scale was evident by the plausible association of professional breast cancer experience with knowledge of breast cancer risk factors. The reliability coefficient (KR-20) of the instrument was unsatisfactorily low. Internal consistency reliability of General Knowledge sub-scale of the Stager's Comprehensive Breast Cancer Knowledge Test was 0.6 [20], but reliability coefficient of our tool cannot be directly compared with this as we have modified the tool. In addition, the low reliability for the present study could be indicative of the vast differences that exist among women in USA, where the Stager's tool was validated, and the Pakistani nurses. Also Stager's General Knowledge sub-scale was adapted for a survey among Jordanian nurses [18] and a low reliability coefficient of 0.26 was reported. Our tool needs to be revised in a reliability study to improve the internal consistency.

## Conclusion

The level of good knowledge of breast cancer risk factors among female registered nurses working in teaching hospitals of Karachi was low (35%). The private affiliation of school of nursing and ever having cared for a breast cancer patient had a positive influence on the knowledge of risk factors.

There is a need to improve breast cancer content in the nursing curriculum. As the implementation of the revised curriculum may take some time, workplace training courses for the nurses can be introduced relatively earlier. It is also important to encourage the nurses to disseminate this knowledge effectively and appropriately within the general population.

Similar studies among health professionals in other parts of Pakistan could provide evidence that will facilitate a better understanding of the level of awareness of breast cancer within the Pakistani health community.

## Competing interests

The author(s) declare that they have no competing interests.

## Authors' contributions

FA did the study under the supervision of SM and performed the statistical analysis and drafted the manuscript. JH provided methodological input including statistical expertise. SK provided clinical knowledge of breast cancer and prevailing status of breast cancer awareness among allied health professionals. All authors have reviewed the final manuscript.

## Pre-publication history

The pre-publication history for this paper can be accessed here:


